# Results from the Survey of Antibiotic Resistance (SOAR) 2018–21 in Ukraine: data based on CLSI, EUCAST (dose-specific) and pharmacokinetic/pharmacodynamic (PK/PD) breakpoints

**DOI:** 10.1093/jac/dkaf287

**Published:** 2025-11-24

**Authors:** Didem Torumkuney, Elena Bratus, Olena Yuvko, Tetyana Pertseva, Ian Morrissey, Cristiana Ossaille Beltrame, Anand Manoharan

**Affiliations:** Infectious Disease Research Unit, GSK, London, UK; Diagnostic Center, Dnipropetrovsk State Medical Academy, Dnipropetrovsk, Kyiv, Ukraine; Diagnostic Center, Dnipropetrovsk State Medical Academy, Dnipropetrovsk, Kyiv, Ukraine; Medical Science, Dnipropetrovsk State Medical Academy, Dnipro, Ukraine; Antimicrobial Focus Ltd., Sawbridgeworth, UK; a Departamento de Microbiologia Médica, Instituto de Microbiologia Paulo de Góes, Universidade Federal do Rio de Janeiro, Rio de Janeiro, Brazil; Infectious Diseases Medical & Scientific Affairs, GSK, Mumbai, India

## Abstract

**Objectives:**

To determine the antibiotic susceptibility of *Streptococcus pneumoniae* and *Haemophilus influenzae* from community-acquired respiratory tract infections (CA-RTIs) collected in 2018–21 from Ukraine.

**Methods:**

MICs were determined by CLSI broth microdilution test, and susceptibility data were interpreted using CLSI, EUCAST (dose-specific) and pharmacokinetic/pharmacodynamic (PK/PD) breakpoints.

**Results:**

*S. pneumoniae* (*n* = 64) and *H. influenzae* (*n* = 76) isolates were collected in 2018–21. Antibiotic susceptibility was 82.8% for pneumococci penicillin-susceptible by CLSI oral/EUCAST low-dose breakpoints and 98.4% by EUCAST high-dose/CLSI intravenous breakpoints. Fluoroquinolones were the most active (100% susceptible). CLSI susceptibility of 85.9%–96.9% was also observed for amoxicillin, amoxicillin/clavulanic acid and cephalosporins. Tetracycline and macrolide susceptibility was 73.4%–76.6%, and trimethoprim/sulfamethoxazole was 59.4%. EUCAST susceptibility was similar if high-dose regimens were chosen, although susceptibility to trimethoprim/sulfamethoxazole was 79.7%. Most *H. influenzae* isolates were β-lactamase negative (*n* = 72, 94.7%) with >94.7% susceptibility, except for trimethoprim/sulfamethoxazole (61.8% by CLSI/EUCAST low-dose, 68.4% by EUCAST high-dose). Susceptibility using EUCAST breakpoints was similar to CLSI, except cefuroxime (oral) with 0% of isolates susceptible versus 100% by CLSI. Cefaclor susceptibility was 11.8% using PK/PD breakpoints.

**Conclusions:**

For *H. influenzae*, 100% susceptibility (CLSI) was observed to amoxicillin/clavulanic acid, macrolides, most cephalosporins and tetracycline. *S. pneumoniae* susceptibility was 100% to fluoroquinolones, 90.6%–96.9% to third-generation cephalosporins and 96.9% to amoxicillin and amoxicillin/clavulanic acid. This is consistent with previous resistance surveillance in Ukraine. Continued surveillance of antibiotic susceptibility is important for guiding empiric therapy of CA-RTIs.

## Introduction

Community-acquired respiratory tract infections (CA-RTIs) are an important world health problem that, when treated inappropriately, can result in hospitalization, with a third of patients with community-acquired pneumonia dying within 12 months after being discharged from hospital.^1^ Factors such as comorbidities, age and other risk factors may have also impacted the mortality rate.^1^ Treatment of CA-RTIs is reliant on empiric antibiotic therapy following national and international guidelines.^2^

*Streptococcus pneumoniae* and *Haemophilus influenzae* are the main bacteria associated with CA-RTIs.^3,4^ Both pathogens have shown increasing resistance to first-line antibiotics such as penicillin and ampicillin.^5,6^ As rates of resistance vary over time, and between countries, up-to-date surveillance data are essential to guide local antibiotic policies.^7^

The Survey of Antibiotic Resistance (SOAR), an international antibiotic resistance surveillance study, focuses on key respiratory pathogens that cause community-acquired infections. SOAR has been running since 2002 in the Middle East, Africa, Latin America, Asia-Pacific, Europe and the Commonwealth of Independent States countries.^8^ SOAR surveillance in Ukraine has been ongoing since 2011. In this study, recent SOAR data from one hospital in Ukraine that has been consistently involved in SOAR surveillance have been analysed to provide a picture of the current state of antibiotic susceptibility of *S. pneumoniae* and *H. influenzae* associated with CA-RTIs.

## Materials and methods

### Ethics

SOAR studies are not human subject studies. During the study, only microorganisms were examined.

### Clinical isolates

Isolates of *H. influenzae* and *S. pneumoniae* from CA-RTIs (isolated within 48 h of hospitalization) were collected between 2018 and 2021 from the Dnipro State Medical University, Dnipro, Ukraine, and sent to a central laboratory (IHMA Europe, Monthey, Switzerland), where they were sub-cultured. *H. influenzae* were re-identified by MALDI-TOF MS methodology, and identification of *S. pneumoniae* was confirmed by optochin susceptibility and bile solubility. β-Lactamase production for *H. influenzae* was determined by a chromogenic cephalosporin (nitrocefin) disc method. Duplicate isolates from the same patient were not accepted.

### Susceptibility testing

Antibiotic susceptibility of isolates was evaluated using broth microdilution methodology recommended by CLSI.^9^ Amoxicillin, amoxicillin/clavulanic acid (2:1 ratio as per CLSI guidelines^9,10^), amoxicillin/clavulanic acid (fixed clavulanic acid at 2 mg/L as per EUCAST guidelines^11^), azithromycin, cefaclor, cefdinir, cefixime, cefotaxime, cefpodoxime, ceftibuten, ceftriaxone, cefuroxime, clarithromycin, levofloxacin, moxifloxacin and trimethoprim/sulfamethoxazole (1:19 ratio) were tested against both respiratory pathogens. Additionally, doxycycline, erythromycin and penicillin were tested against *S. pneumoniae* only, while ampicillin was tested against *H. influenzae* only. Susceptibility to the study drugs was calculated based on CLSI breakpoints, EUCAST (dose-specific) breakpoints and pharmacokinetic/pharmacodynamic (PK/PD) breakpoints.^10–12^ These breakpoints are shown in Tables 1–3. To fully assess antibiotics where high-dose therapies are available, susceptibility using EUCAST criteria was also calculated by combining percentage susceptible and susceptible, increased exposure into the susceptible category.^11^ The antibiotics with high-dose availability assessed in this way included the following: amoxicillin (0.75–1 g oral, 3× daily), amoxicillin/clavulanic acid (0.875 g amoxicillin/0.125 g clavulanic acid oral, 3× daily), ampicillin [2 g intravenous (IV), 4×daily], penicillin (2.4 g IV, 2 MU 4–6× daily), ceftriaxone (2 g IV, 2× daily), clarithromycin (0.5 g oral, 2× daily), erythromycin (1 g oral or IV, 4× daily), levofloxacin (0.75 g oral 2× daily, or 0.4 g IV 3× daily) and trimethoprim/sulfamethoxazole (0.24 g trimethoprim/1.2 g sulfamethoxazole oral or IV, 2× daily).^11^

**Table 1.** CLSI MIC breakpoints (mg/L) used for *S. pneumoniae* and *H. influenzae* isolates

	*S. pneumoniae*			*H. influenzae*		
Antimicrobial	S	I	R	S	I	R
Amoxicillin	≤2	4	≥8	—	—	—
Amoxicillin/clavulanic acid (2:1)^a^	≤2	4	≥8	≤2	4	≥8
Ampicillin	NT	NT	NT	≤1	2	≥4
Azithromycin	≤0.5	1	≥2	≤4	—	—
Cefaclor	≤1	2	≥4	≤8	16	≥32
Cefdinir	≤0.5	1	≥2	≤1	—	—
Cefixime	—	—	—	≤1	—	—
Cefotaxime (non-meningitis)	≤1	2	≥4	≤2	—	—
Cefpodoxime	≤0.5	1	≥2	≤2	—	—
Ceftibuten	—	—	—	≤2	—	—
Ceftriaxone (non-meningitis)	≤1	2	≥4	≤2	—	—
Cefuroxime^b^	≤1	2	≥4	≤4	8	≥16
Clarithromycin	≤0.25	0.5	≥1	≤8	16	≥32
Doxycycline	≤0.25	0.5	≥1	NT	NT	NT
Erythromycin	≤0.25	0.5	≥1	NT	NT	NT
Levofloxacin	≤2	4	≥8	≤2	—	—
Moxifloxacin	≤1	2	≥4	≤1	—	—
Penicillin (2.4 g 2 MU × 4–6 IV)	≤2	4	≥8	NT	NT	NT
Penicillin (oral)	≤0.06	0.12–1	≥2	NT	NT	NT
Tetracycline	≤1	2	≥4	≤2	4	≥8
Trimethoprim/sulfamethoxazole^c^	≤0.5	1–2	≥4	≤0.5	1–2	≥4

—, not applicable; I, intermediate; NT, not tested; R, resistant; S, susceptible.

^a^Amoxicillin/clavulanic acid was tested at a 2:1 amoxicillin to clavulanic acid ratio; breakpoints are expressed as the amoxicillin component.

^b^Breakpoints used are for cefuroxime axetil (oral).

^c^Trimethoprim/sulfamethoxazole was tested at a 1:19 trimethoprim to sulfamethoxazole ratio; breakpoints are expressed as the trimethoprim component.

**Table 2.** EUCAST (dose-specific) MIC breakpoints (mg/L) used for *S. pneumoniae* and *H. influenzae* isolates

	*S. pneumoniae*		*H. influenzae*	
Antimicrobial^a^	S	R	S	R
Amoxicillin (0.5 g × 3 oral)	≤0.5	>1	≤0.001	>2
Amoxicillin (0.75–1 g × 3 oral)	≤1	>1	≤2	>2
Amoxicillin/clavulanic acid (0.5 g/0.125 g × 3 oral)^b^	≤0.5	>1	≤0.001	>2
Amoxicillin/clavulanic acid (0.875 g/0.125 g × 3 oral)^b^	≤1	>1	≤2	>2
Ampicillin (2 g × 3 IV)	NT	NT	≤1	>1
Ampicillin (2 g × 4 IV)	NT	NT	≤1	>1
Azithromycin	≤0.25	>0.5	—	—
Cefaclor	≤0.001	>0.5	—	—
Cefdinir	—	—	—	—
Cefixime	—	—	≤0.12	>0.12
Cefotaxime	≤0.5	>2	≤0.12	>0.12
Cefpodoxime	≤0.25	>0.5	≤0.25	>0.25
Ceftibuten	—	—	≤1	>1
Ceftriaxone (1 g × 1 IV)	≤0.5	>2	≤0.12	>0.12
Ceftriaxone (2 g × 2 IV)	≤2	>2	≤0.12	>0.12
Cefuroxime^c^	≤0.25	>0.5	≤0.001	>1
Clarithromycin (0.25 g × 2 oral)	≤0.25	>0.5	—	—
Clarithromycin (0.5 g × 2 oral)	≤0.5	>0.5	—	—
Doxycycline	≤1	>2	NT	NT
Erythromycin (0.5 g × 2–4 oral or 0.5 g × 2–4 IV)	≤0.25	>0.5	NT	NT
Erythromycin (1 g × 4 oral or 1 g × 4 IV)	≤0.5	>0.5	NT	NT
Levofloxacin (0.5 g × 2 oral or 0.4 g × 2 IV)	≤0.001	>2	≤0.06	>0.06
Levofloxacin (0.75 g × 2 oral or 0.4 g × 3 IV)	≤2	>2	≤0.06	>0.06
Moxifloxacin	≤0.5	>0.5	≤0.12	>0.12
Penicillin (0.6 g 1 MU × 4 IV)	≤0.06	>2	NT	NT
Penicillin (2.4 g 2 MU × 4–6 IV)	≤2	>2	NT	NT
Tetracycline	≤1	>2	≤2	>2
Trimethoprim/sulfamethoxazole (0.16 g/0.8 g × 2 oral or IV)^d^	≤1	>2	≤0.5	>1
Trimethoprim/sulfamethoxazole (0.24 g/1.2 g × 2 oral or IV)^d^	≤2	>2	≤1	>1

—, not applicable; S, susceptible; R, resistant; NT, not tested.

^a^Where available, susceptibility was assessed using EUCAST higher dosage breakpoints.

^b^Amoxicillin/clavulanic acid was tested at a fixed concentration of 2 mg/L; breakpoints are expressed as the amoxicillin component.

^c^Breakpoints used are for cefuroxime axetil (oral).

^d^Trimethoprim/sulfamethoxazole was tested at a 1:19 trimethoprim to sulfamethoxazole ratio; breakpoints are expressed as the trimethoprim component.

**Table 3.** PK/PD MIC breakpoints (mg/L) used for *S. pneumoniae* and *H. influenzae* isolates

	*S. pneumoniae* and *H. influenzae*
Antimicrobial	S only
Amoxicillin (1.5 g/day)^a^	≤2
Amoxicillin (4 g/day)^b^	≤4
Amoxicillin/clavulanic acid^a^ (1.75 g/0.25 g/day adults; 45 mg/6.4 mg/kg/day children)	≤2
Amoxicillin/clavulanic acid^b^ (4 g/0.25 g/day adults; 90 mg/6.4 mg/kg/day children)	≤4
Ampicillin	—
Penicillin	—
Cefaclor	≤0.5
Cefdinir	≤0.25
Cefditoren	—
Cefixime	≤1
Cefpodoxime	≤0.5
Ceftriaxone	≤1
Cefuroxime^c^	≤1
Azithromycin	≤0.12
Clarithromycin	≤0.25
Erythromycin	≤0.25
Levofloxacin	≤2
Moxifloxacin	≤1
Trimethoprim/sulfamethoxazole^d^	≤0.5

—, not applicable; PK/PD, pharmacokinetic/pharmacodynamic; S, susceptible.

^a^Amoxicillin/clavulanic acid for low dose in adults/children.

^b^Amoxicillin/clavulanic acid for high dose in adults/children.

^c^Breakpoints used are for cefuroxime axetil (oral).

^d^Trimethoprim/sulfamethoxazole was tested at a 1:19 trimethoprim to sulfamethoxazole ratio; breakpoints are expressed as the trimethoprim component.

### Quality control and data analysis

Quality control strains *S. pneumoniae* ATCC 49619, *H. influenzae* ATCC 49247, *H. influenzae* ATCC 49766 and *E. coli* ATCC 32518 were included on each day of testing. Results of susceptibility testing were only accepted if the results of the quality control strains were within the published acceptable range. Differences in susceptibility (using CLSI criteria only) were assessed for statistical significance using the Fisher’s exact test with XLSTAT version 2023.1.1.1399 for isolates from this study period (2018–21) compared with SOAR data from Ukraine 2015–17.^13^ A *P* < 0.05 was considered statistically significant.

## Results

### *S. pneumoniae* isolates

A total of 64 *S. pneumoniae* isolates were collected between 2018 and 2021. Most isolates came from sputum (*n* = 34, 53.1%), with the remainder from sinus (*n* = 14, 21.9%), blood (*n* = 8, 12.5%), bronchoalveolar lavage (*n* = 5, 7.8%), middle ear (*n* = 2, 3.1%) and unidentified specimens (*n* = 1, 1.6%). The majority of isolates (*n* = 45, 70.3%) came from adolescent and adult patients (aged 13–64 years), and 11 (17.2%) isolates were from elderly patients (aged ≥65 years) and 8 (12.5%) isolates from paediatric patients (aged ≤12 years).

**Figure 1.**
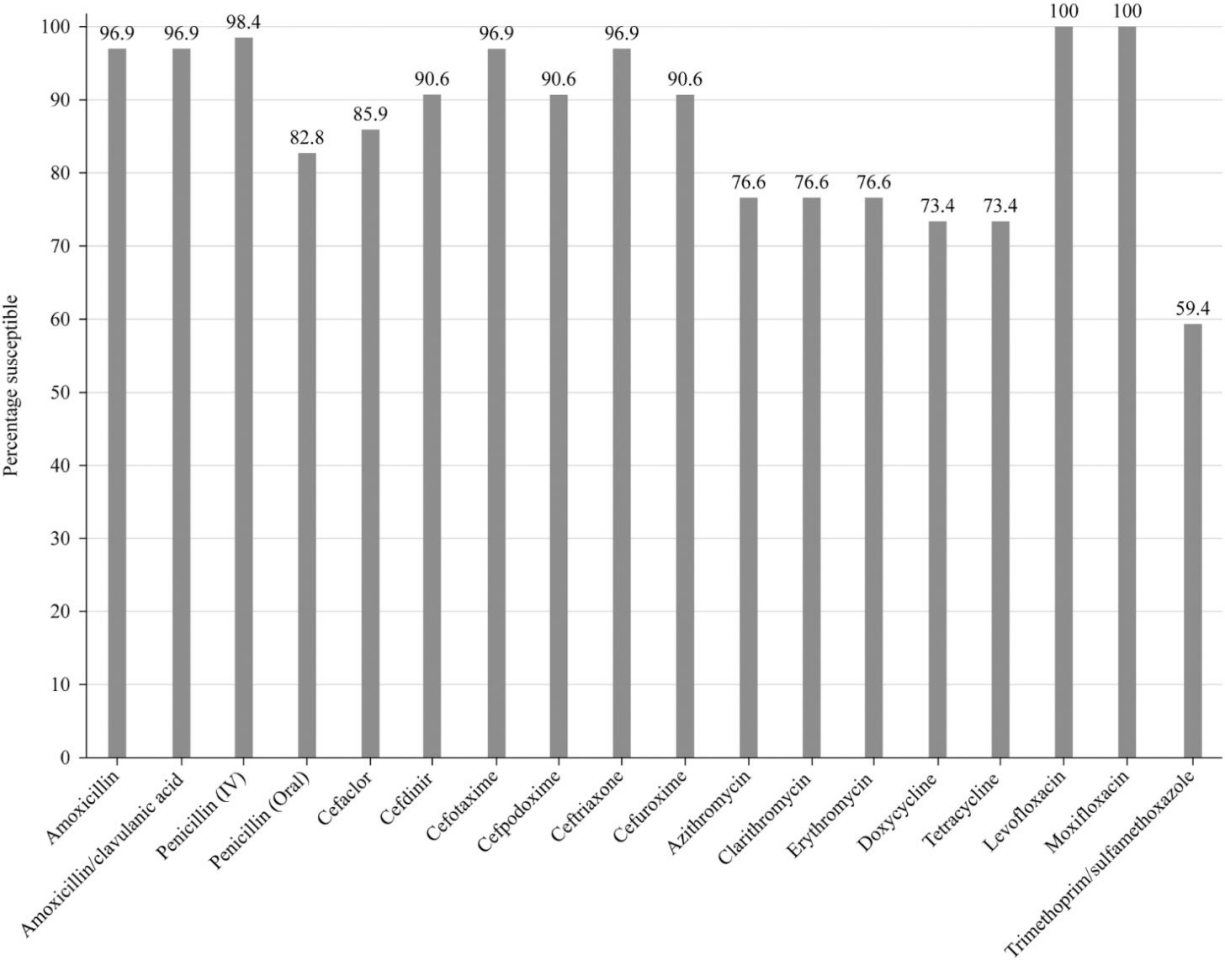
Antibiotic susceptibility rates of *S. pneumoniae* isolates (*n* = 64) from Ukraine based on CLSI breakpoints.

**Figure 2.**
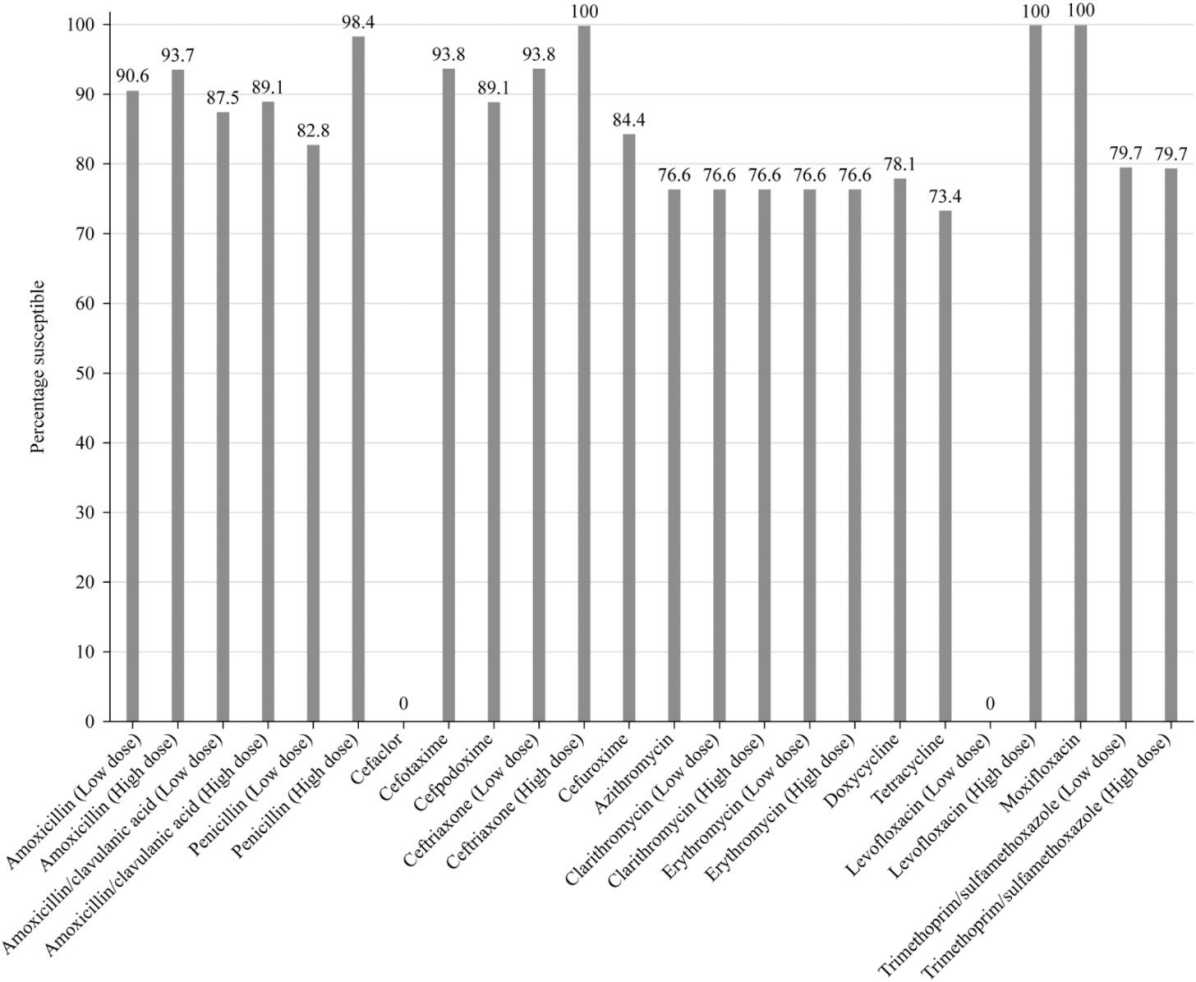
Antibiotic susceptibility rates of *S. pneumoniae* isolates (*n* = 64) from Ukraine based on EUCAST (dose-specific) breakpoints.

Summary MIC, susceptibility and MIC distribution data for all 64 *S. pneumoniae* isolates are given in Tables 4–6 and S1 (available as Supplementary data at *JAC* Online) and shown in Figures 1 and 2.

**Table 4.** MIC and susceptibility data for *S. pneumoniae* isolates (*n* = 64) from Ukraine using CLSI breakpoints

	MIC (mg/L)			CLSI susceptibility		
Antimicrobial	Range	50%	90%	%S	%I	%R
Amoxicillin	0.015–8	0.015	0.5	96.9	1.6	1.6
Amoxicillin/clavulanic acid (2:1)	≤0.008–8	0.03	0.5	96.9	1.6	1.6
Penicillin (2.4 g 2 MU × 4–6 IV)	≤0.008–4	0.015	0.25	98.4	1.6	0
Penicillin (oral)	≤0.008–4	0.015	0.25	82.8	10.9	6.3
Cefaclor	0.06–>4	0.5	>4	85.9	3.1	10.9
Cefdinir	0.03–>8	0.06	0.5	90.6	1.6	7.8
Cefixime	≤0.25–>16	≤0.25	4	—	—	—
Cefotaxime	≤0.008–2	0.03	0.25	96.9	3.1	0
Cefpodoxime	≤0.015–4	0.03	0.5	90.6	3.1	6.3
Ceftibuten	2–>16	4	>16	—	—	—
Ceftriaxone	0.015–2	0.03	0.25	96.9	3.1	0
Cefuroxime	0.015–>8	0.03	1	90.6	1.6	7.8
Azithromycin	≤0.015–>16	0.06	>16	76.6	0	23.4
Clarithromycin	≤0.015–>16	0.03	>16	76.6	0	23.4
Erythromycin	≤0.015–>16	0.03	>16	76.6	0	23.4
Doxycycline	0.03–>4	0.06	>4	73.4	3.1	23.4
Tetracycline	0.06–>4	0.25	>4	73.4	0	26.6
Levofloxacin	0.5–2	1	2	100	0	0
Moxifloxacin	0.06–0.25	0.12	0.12	100	0	0
Trimethoprim/sulfamethoxazole	0.12–>8	0.5	8	59.4	20.3	20.3

—, not applicable; I, intermediate; R, resistant; S, susceptible.

**Table 5.** MIC and susceptibility data for *S. pneumoniae* isolates (*n* = 64) from Ukraine using EUCAST (dose-specific) breakpoints

	MIC (mg/L)			EUCAST susceptibility		
Antimicrobial	Range	50%	90%	%S	%I	%R
Amoxicillin (0.5 g × 3 oral)	0.015–8	0.015	0.5	90.6	3.1	6.3
Amoxicillin (0.75–1 g × 3 oral)	0.015–8	0.015	0.5	93.7	—	6.3
Amoxicillin/clavulanic acid (0.5 g/0.125 g × 3 oral)	0.03–>8	0.06	2	87.5	1.6	10.9
Amoxicillin/clavulanic acid (0.875 g/0.125 g × 3 oral)	0.03–>8	0.06	2	89.1	—	10.9
Penicillin (0.6 g 1 MU × 4 IV)	≤0.008–4	0.015	0.25	82.8	15.6	1.6
Penicillin (2.4 g 2 MU × 4–6 IV)	≤0.008–4	0.015	0.25	98.4	—	1.6
Cefaclor	0.06–>4	0.5	>4	0	82.8	17.2
Cefdinir	0.03–>8	0.06	0.5	—	—	—
Cefixime	≤0.25–>16	≤0.25	4	—	—	—
Cefotaxime	≤0.008–2	0.03	0.25	93.8	6.3	0
Cefpodoxime	≤0.015–4	0.03	0.5	89.1	1.6	9.4
Ceftibuten	2–>16	4	>16	—	—	—
Ceftriaxone (1 g × 1 IV)	0.015–2	0.03	0.25	93.8	6.3	0
Ceftriaxone (2 g × 2 IV)	0.015–2	0.03	0.25	100	—	0
Cefuroxime	0.015–>8	0.03	1	84.4	3.1	12.5
Azithromycin	≤0.015–>16	0.06	>16	76.6	—	23.4
Clarithromycin (0.25 g × 2 oral)	≤0.015–>16	0.03	>16	76.6	0	23.4
Clarithromycin (0.5 g × 2 oral)	≤0.015–>16	0.03	>16	76.6	—	23.4
Erythromycin (0.5 g × 2–4 oral or 0.5 g × 2–4 IV)	≤0.015–>16	0.03	>16	76.6	0	23.4
Erythromycin (1 g × 4 oral or 1 g × 4 IV)	≤0.015–>16	0.03	>16	76.6	—	23.4
Doxycycline	0.03–>4	0.06	>4	78.1	1.6	20.3
Tetracycline	0.06–>4	0.25	>4	73.4	0	26.6
Levofloxacin (0.5 g × 2 oral or 0.4 g × 2 IV)	0.5–2	1	2	0	100	0
Levofloxacin (0.75 g × 2 oral or 0.4 g × 3 IV)	0.5–2	1	2	100	—	0
Moxifloxacin	0.06–0.25	0.12	0.12	100	—	0
Trimethoprim/sulfamethoxazole (0.16 g/0.8 g × 2 oral or IV)	0.12–>8	0.5	8	79.7	0	20.3
Trimethoprim/sulfamethoxazole (0.24 g/1.2 g × 2 oral or IV)	0.12–>8	0.5	8	79.7	—	20.3

—, not applicable; S, susceptible; I, susceptible, increased exposure; R, resistant.

**Table 6.** Summary MIC and susceptibility data for *S. pneumoniae* (*n* = 64) from Ukraine using PK/PD breakpoints

	MIC (mg/L)			PK/PD susceptibility
Antimicrobial	Range	50%	90%	%S
Amoxicillin (1.5 g/day)	0.015–8	0.015	0.5	96.9
Amoxicillin (4 g/day)	0.015–8	0.015	0.5	98.4
Amoxicillin/clavulanic acid (1.75 g/0.25 g/day adults; 45 mg/6.4 mg/kg/day children)	≤0.008–8	0.03	0.5	96.9
Amoxicillin/clavulanic acid (4 g/0.25 g/day adults; 90 mg/6.4 mg/kg/day children)	≤0.008–8	0.03	0.5	98.4
Penicillin	≤0.008–4	0.015	0.25	—
Cefaclor	0.06–>4	0.5	>4	82.8
Cefdinir	0.03–>8	0.06	0.5	87.5
Cefixime	≤0.25–>16	≤0.25	4	81.3
Cefotaxime	≤0.008–2	0.03	0.25	—
Cefpodoxime	≤0.015–4	0.03	0.5	90.6
Ceftibuten	2–>16	4	>16	—
Ceftriaxone	0.015–2	0.03	0.25	96.9
Cefuroxime	0.015–>8	0.03	1	90.6
Azithromycin	≤0.015–>16	0.06	>16	75.0
Clarithromycin	≤0.015–>16	0.03	>16	76.6
Erythromycin	≤0.015–>16	0.03	>16	76.6
Doxycycline	0.03–>4	0.06	>4	73.4
Tetracycline	0.06–>4	0.25	>4	—
Levofloxacin	0.5–2	1	2	100
Moxifloxacin	0.06–0.25	0.12	0.12	100
Trimethoprim/sulfamethoxazole	0.12–>8	0.5	8	59.4

—, not applicable; PK/PD, pharmacokinetic/pharmacodynamic; S, susceptible.

### *S. pneumoniae* susceptibility

Overall, antibiotic susceptibility of pneumococci in Ukraine was 82.8% to penicillin when CLSI oral or EUCAST low-dose (0.6 g 1 MU × 4 IV) breakpoints were applied. If EUCAST high-dose (2.4 g 2 MU × 4–6 IV) or CLSI IV breakpoints were used, susceptibility increased to 98.4%. According to CLSI breakpoints, amoxicillin and amoxicillin/clavulanic acid susceptibility was 96.9% and cephalosporin susceptibility ranged from 85.9% (cefaclor) to 96.9% (ceftriaxone and cefotaxime). Susceptibility according to PK/PD breakpoints was very similar to CLSI, except higher dosing for amoxicillin (4 g/day) and amoxicillin/clavulanic acid (4 g/0.25 g/day) increased susceptibility to 98.4% for both. EUCAST breakpoints for amoxicillin and amoxicillin/clavulanic acid were more conservative resulting in slightly lower susceptibility (87.5%–93.7%) than that obtained by CLSI breakpoints depending on the dosing regimen. Susceptibility by EUCAST breakpoints was also slightly lower than that obtained using CLSI breakpoints for cefotaxime, cefpodoxime, cefuroxime and ceftriaxone. However, 0% susceptibility for cefaclor was obtained using EUCAST breakpoints compared with 85.9% by CLSI and 82.8% by PK/PD breakpoints. Less activity was observed for the macrolides (azithromycin, clarithromycin and erythromycin) and tetracyclines (doxycycline and tetracycline) by CLSI, EUCAST or PK/PD interpretation (73.4%–76.6% susceptibility). Similar activity was observed for trimethoprim/sulfamethoxazole (79.7% susceptibility) using EUCAST breakpoints, but activity was reduced using CLSI and PK/PD breakpoints (59.4% susceptible). Full susceptibility to moxifloxacin was observed using any one of the three breakpoints, and levofloxacin susceptibility was also 100% by CLSI and PK/PD. However, only the high-dose regimen (0.75 g × 2 oral or 0.4 g × 3 IV) achieved this level of activity following EUCAST breakpoints (Tables 4–6 and S1 and Figures 1 and 2).

### Comparative susceptibility of *S. pneumoniae* collected in 2016–17 and 2018–21

**Figure 3.**
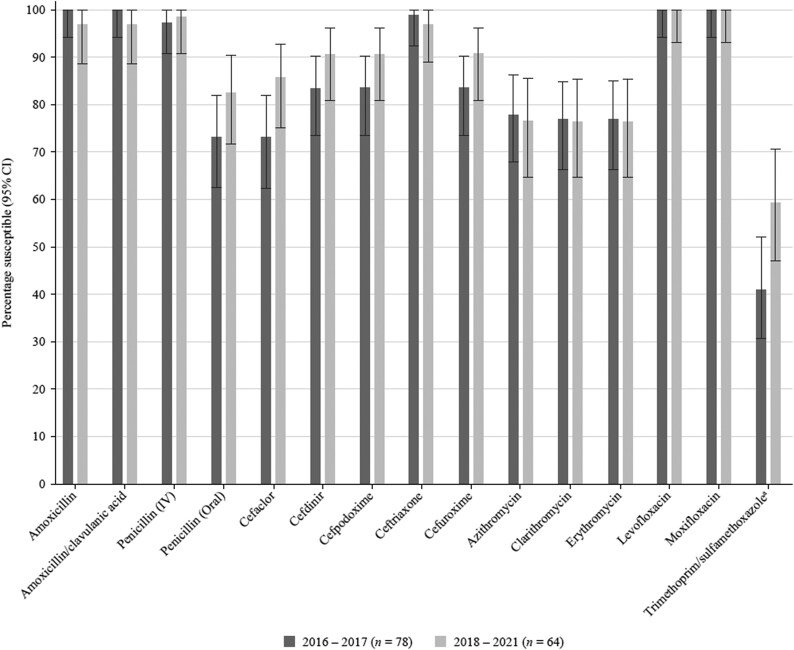
Comparison of antibiotic susceptibility rates of *S. pneumoniae* isolates from Ukraine collected in 2016–17 with isolates collected in 2018–21 (CLSI breakpoints). ^a^Susceptibility was significantly higher in 2018–21 than 2016–17 (*P* = 0.043). CI, confidence interval.

Data have previously been published from the SOAR surveillance for the period 2016–17, and data were compared for mutually tested antibiotics with the current study (2018–21) (Figure 3). As would be expected from the generally high susceptibility of pneumococci from Ukraine to antibiotics, there was no significant change in susceptibility except for an increased level of susceptibility to trimethoprim/sulfamethoxazole (41.0% versus 59.4%).

### *H. influenzae* isolates

**Figure 4.**
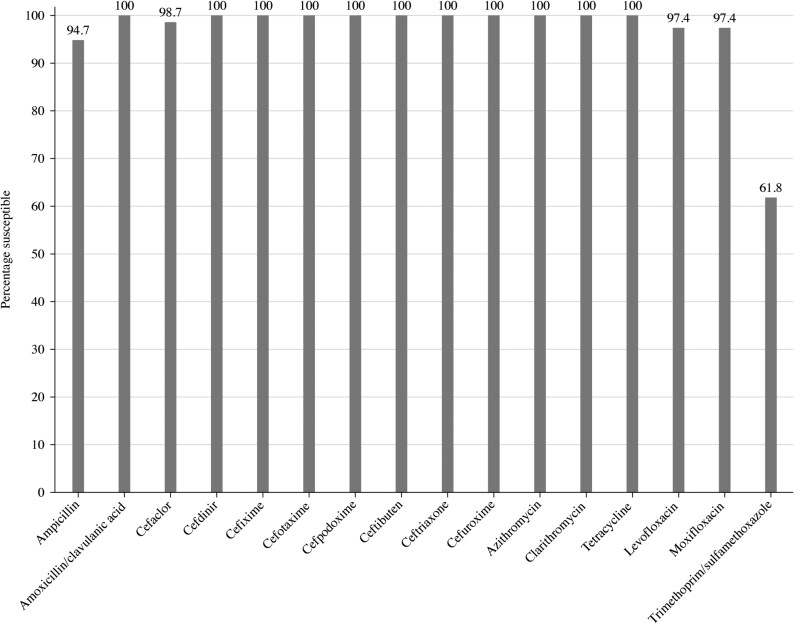
Antibiotic susceptibility rates of *H. influenzae* isolates (*n* = 76) from Ukraine based on CLSI breakpoints.

**Figure 5.**
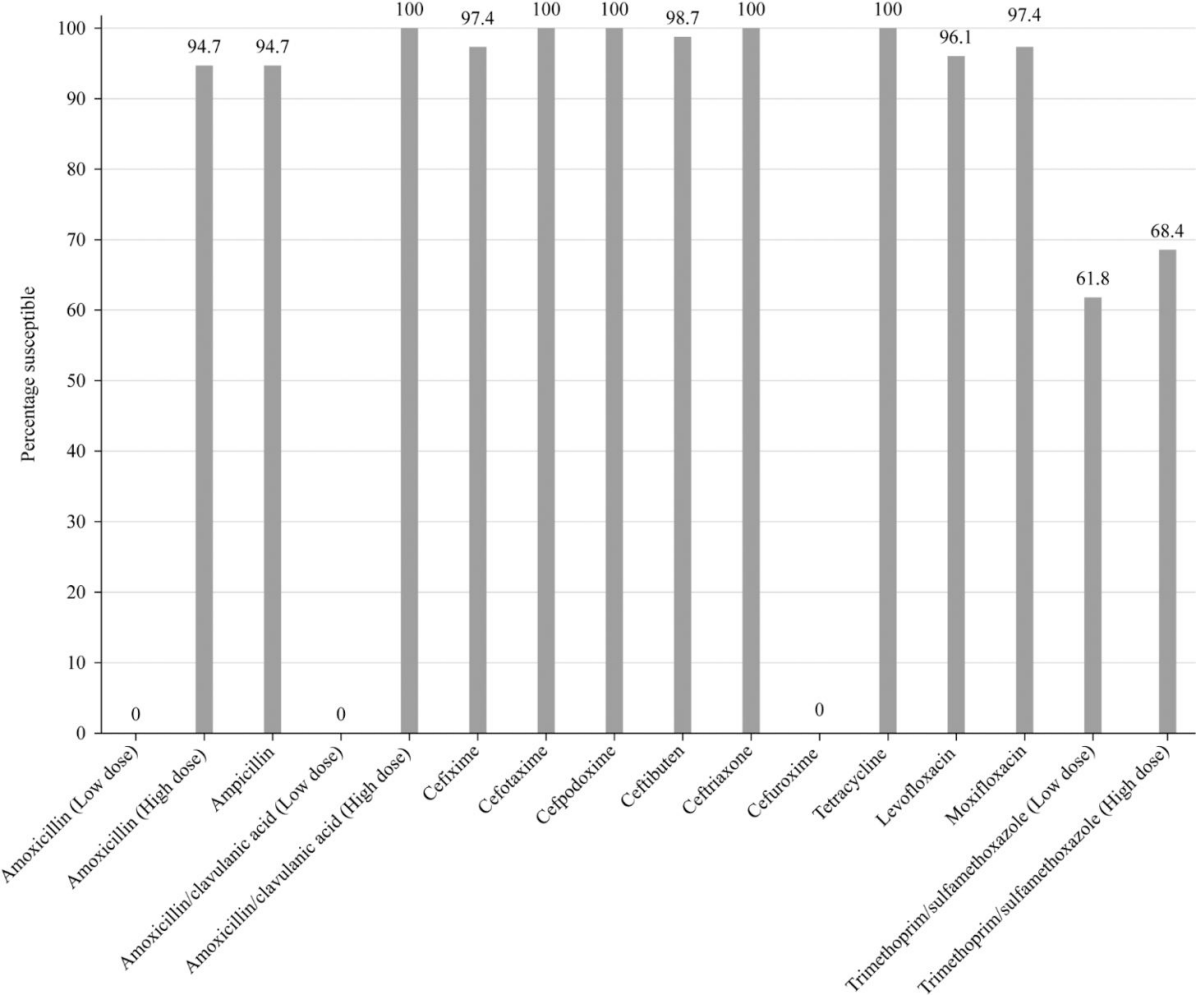
Antibiotic susceptibility rates of *H. influenzae* isolates (*n* = 76) from Ukraine based on EUCAST (dose-specific) breakpoints.

A total of 76 *H. influenzae* isolates were collected in Ukraine from 2018 to 2021. Most isolates originated from sputum (*n* = 60; 78.9%). The remaining isolates were from sinus (*n* = 11; 14.5%) and bronchoalveolar lavage (*n* = 5; 6.6%). Just over two-thirds of the isolates (*n* = 51; 67.1%) came from adolescents and adults (aged 13–64 years), while isolates from elderly patients (aged ≥65 years) represented 27.6% (*n* = 21) and the remaining 5.3% (*n* = 4) were from paediatric patients (aged ≤12 years). Summary MIC, susceptibility and MIC distribution data for all 76 *H. influenzae* isolates are given in Tables 7–9 and S2 and shown in Figures 4 and 5.

**Table 7.** MIC and susceptibility data for *H. influenzae* isolates (*n* = 76) from Ukraine using CLSI breakpoints

	MIC (mg/L)			CLSI susceptibility		
Antimicrobial	Range	50%	90%	%S	%I	%R
Amoxicillin	0.12–64	0.25	1	—	—	—
Ampicillin	0.12–128	0.12	1	94.7	0	5.3
Amoxicillin/clavulanic acid (2:1)	0.12–2	0.25	1	100	0	0
Cefaclor	≤0.25–16	1	4	98.7	1.3	0
Cefdinir	0.12–1	0.25	0.5	100	—	—
Cefixime	≤0.008–0.25	0.03	0.03	100	—	—
Cefotaxime	0.004–0.06	0.008	0.03	100	—	—
Cefpodoxime	≤0.015–0.25	0.03	0.12	100	—	—
Ceftibuten	0.03–2	0.06	0.12	100	—	—
Ceftriaxone	0.002–0.015	0.004	0.008	100	—	—
Cefuroxime	0.25–4	0.5	1	100	0	0
Azithromycin	0.25–2	0.5	1	100	—	—
Clarithromycin	2–8	4	8	100	0	0
Tetracycline	0.25–0.5	0.5	0.5	100	—	—
Levofloxacin	0.008–8	0.015	0.015	97.4	—	—
Moxifloxacin	≤0.004–8	0.015	0.03	97.4	—	—
Trimethoprim/sulfamethoxazole	0.03–>8	0.12	8	61.8	11.8	26.3

—, not applicable; I, intermediate; R, resistant; S, susceptible.

**Table 8.** MIC and susceptibility data for *H. influenzae* isolates (*n* = 76) from Ukraine using EUCAST (dose-specific) breakpoints

	MIC (mg/L)			EUCAST susceptibility		
Antimicrobial	Range	50%	90%	%S	%I	%R
Amoxicillin (0.5 g × 3 oral)	0.12–64	0.25	1	0	94.7	5.3
Amoxicillin (0.75–1 g × 3 oral)	0.12–64	0.25	1	94.7	0	5.3
Ampicillin	0.12–128	0.12	1	94.7	0	5.3
Amoxicillin/clavulanic acid (0.5 g/0.125 g × 3 oral)	0.12–2	0.25	0.5	0	100	0
Amoxicillin/clavulanic acid (0.875 g/0.125 g × 3 oral)	0.12–2	0.25	0.5	100	0	0
Cefaclor	≤0.25–16	1	4	—	—	—
Cefdinir	0.12–1	0.25	0.5	—	—	—
Cefixime	≤0.008–0.25	0.03	0.03	97.4	0	2.6
Cefotaxime	≤0.002–0.06	0.008	0.03	100	0	0
Cefpodoxime	≤0.015–0.25	0.03	0.12	100	0	0
Ceftibuten	0.03–2	0.06	0.12	98.7	0	1.3
Ceftriaxone	≤0.001–0.015	0.004	0.008	100	0	0
Cefuroxime	0.25–4	0.5	1	0	92.1	7.9
Azithromycin	0.25–2	0.5	1	—	—	—
Clarithromycin	2–8	4	8	—	—	—
Tetracycline	0.25–0.5	0.5	0.5	100	0	0
Levofloxacin	0.008–>8	0.015	0.015	96.1	0	3.9
Moxifloxacin	≤0.004–8	0.015	0.03	97.4	0	2.6
Trimethoprim/sulfamethoxazole (0.16 g/0.8 g × 2 oral or IV)	0.03–>8	0.12	8	61.8	6.6	31.6
Trimethoprim/sulfamethoxazole (0.24 g/1.2 g × 2 oral or IV)	0.03–>8	0.12	8	68.4	0	31.6

—, not applicable; I, susceptible, increased exposure; R, resistant; S, susceptible.

**Table 9.** Summary MIC and susceptibility data for *H. influenzae* (*n* = 76) from Ukraine using PK/PD breakpoints

	MIC (mg/L)			PK/PD susceptibility
Antimicrobial	Range	50%	90%	%S
Amoxicillin (1.5 g/day)	0.12–64	0.25	1	94.7
Amoxicillin (4 g/day)	0.12–64	0.25	1	94.7
Amoxicillin/clavulanic acid (1.75 g/0.25 g/day adults; 45 mg/6.4 mg/kg/day children)	0.12–2	0.25	1	100
Amoxicillin/clavulanic acid (4 g/0.25 g/day adults; 90 mg/6.4 mg/kg/day children)	0.12–2	0.25	1	100
Ampicillin	0.12–128	0.12	1	—
Cefaclor	≤0.25–16	1	4	11.8
Cefdinir	0.12–1	0.25	0.5	86.8
Cefixime	≤0.008–0.25	0.03	0.03	100
Cefotaxime	0.004–0.06	0.008	0.03	—
Cefpodoxime	≤0.015–0.25	0.03	0.12	100
Ceftibuten	0.03–2	0.06	0.12	—
Ceftriaxone	0.002–0.015	0.004	0.008	100
Cefuroxime	0.25–4	0.5	1	92.1
Azithromycin	0.25–2	0.5	1	0
Clarithromycin	2–8	4	8	0
Tetracycline	0.25–0.5	0.5	0.5	—
Levofloxacin	0.008–8	0.015	0.015	97.4
Moxifloxacin	≤0.004–8	0.015	0.03	97.4
Trimethoprim/sulfamethoxazole	0.03–>8	0.12	8	61.8

—, not applicable; PK/PD, pharmacokinetic/pharmacodynamic; S, susceptible.

### *H. influenzae* susceptibility

Most isolates of *H. influenzae* from Ukraine were β-lactamase negative (*n* = 72; 94.7%). None were found to be β-lactamase negative ampicillin-resistant (BLNAR) or β-lactamase positive but ampicillin-susceptible by EUCAST or CLSI breakpoints. As would be expected, 94.7% of the *H. influenzae* were susceptible to ampicillin (CLSI or EUCAST breakpoints). Amoxicillin breakpoints are not provided by CLSI, but EUCAST breakpoints at high-dose (0.75–1 g × 3 oral) also indicated 94.7% susceptibility, but at low-dose (0.5 g × 3 oral), no isolate would be considered susceptible. Using PK/PD low (1.5 g/day) or high (4 g/day) dose, the susceptibility to amoxicillin was 94.7%. Susceptibility of isolates to amoxicillin/clavulanic acid was 100% by CLSI breakpoints and PK/PD breakpoints. Susceptibility at high-dose (0.875 g/0.125 g × 3 oral) EUCAST breakpoints was also 100% but was 0% at low dose. Full susceptibility was observed for all the cephalosporins according to CLSI breakpoints, except for cefaclor, although this second-generation cephalosporin was still highly active (98.7% susceptibility). Cefaclor susceptibility according to PK/PD breakpoints was 11.8% and susceptibility to cefuroxime and cefdinir was 92.1% and 86.8%, respectively. Similar results were seen with EUCAST breakpoints (although these are not provided for cefaclor or cefdinir), except for cefuroxime (100% by CLSI versus 0% by EUCAST). Macrolide breakpoints are not provided by EUCAST against *H. influenzae*, but full susceptibility was observed for azithromycin and clarithromycin by CLSI breakpoints. In stark contrast, no isolate was susceptible to macrolides according to PK/PD breakpoints. CLSI and EUCAST breakpoint standards showed full susceptibility to tetracycline (no PK/PD breakpoint is available), and all three breakpoints indicated fluoroquinolone susceptibility ≥ 96.1%. Trimethoprim/sulfamethoxazole showed the lowest activity with 61.8% susceptible by CLSI, PK/PD and low-dose (0.16 g/0.8 g × 2 oral or IV) EUCAST breakpoints and 68.4% susceptible by high-dose (0.24 g/1.2 g × 2 oral or IV) EUCAST breakpoints (Tables 7–9 and S2 and Figures 4 and 5).

### Comparative susceptibility of *H. influenzae* collected in 2016–17 and 2018–21

**Figure 6.**
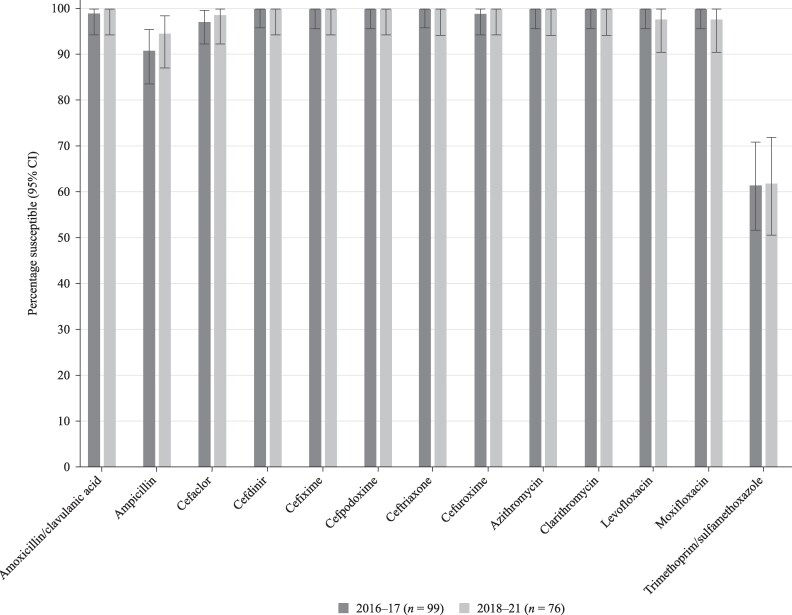
Comparison of antibiotic susceptibility rates of *H. influenzae* isolates from Ukraine collected in 2016–17 with isolates collected in 2018–21 (CLSI breakpoints). CI, confidence interval.

There was no significant change in susceptibility when comparing data from 2016 to 2017 with 2018–21 (Figure 6).

## Discussion

SOAR is an ongoing global surveillance study focusing on the two main CA-RTIs pathogens, *S. pneumoniae* and *H. influenzae*, that has monitored numerous countries since 2002, including Ukraine since 2011. The data presented here are an analysis of the antibiotic susceptibility of *S. pneumoniae* and *H. influenzae* isolates collected from one centre in Ukraine between 2018 and 2021. A single-centre study design may limit the generalizability of the findings at the national level. However, as most isolates originated from community-acquired infections and were presumably unrelated, the findings may be reflective of the broader Ukrainian community. Data from previous SOAR surveillance at this site have been published (2011–13,^14^ 2014–16^15^ and 2016–17^13^). A direct statistical comparison between the published 2016–17 data and the current study is presented here.

High penicillin susceptibility was observed for *S. pneumoniae* from Ukraine with 82.8% susceptibility at the low-dose EUCAST IV or CLSI oral breakpoints and 98.4% using EUCAST high-dose or CLSI IV breakpoints. There was no statistical difference in penicillin susceptibility between this study and the previous SOAR surveillance in Ukraine from 2016 to 2017.^13^ This level of penicillin susceptibility has remained consistent since Ukraine first participated in the SOAR study in 2011.^14^ CLSI breakpoints indicated a similarly high level of susceptibility for amoxicillin, amoxicillin/clavulanic acid, ceftriaxone and cefotaxime (all 96.9%), with other cephalosporins being slightly less active (85.9%–90.6%). Similar results were obtained with PK/PD breakpoints, except higher dose amoxicillin (4 g/day) and amoxicillin/clavulanate (4 g/0.25 g/day) increased susceptibility to 98.4%. When using EUCAST breakpoints, reduced susceptibility was observed compared to CLSI breakpoints for amoxicillin (3.2% lower at EUCAST high-dose) and amoxicillin/clavulanic acid (1.4% lower at EUCAST high-dose), as well as cephalosporins (1.5%–6.2% lower), except for EUCAST high-dose (2 g × 2 IV) breakpoints with ceftriaxone (100% susceptibility). EUCAST breakpoints also consider all *S. pneumoniae* to be non-susceptible to cefaclor. Susceptibility according to both guidelines indicated 73.4%–78.1% susceptibility for macrolides and tetracyclines. Similar trimethoprim/sulfamethoxazole susceptibility (79.7%) was seen by EUCAST breakpoints, but was 59.4% by CLSI breakpoints. Isolates were fully moxifloxacin-susceptible by either guideline and 100% susceptible to levofloxacin by CLSI breakpoints and EUCAST high-dose (0.75 g × 2 oral or 0.4 g × 3 IV) breakpoints.

In this study, we compared the susceptibility of pneumococci using CLSI breakpoints for isolates previously collected in 2016–17 from Ukraine with susceptibility from the current study (2018–21). There was little or no significant difference in susceptibility between the two study periods, except for an increase in trimethoprim/sulfamethoxazole susceptibility. Nevertheless, trimethoprim/sulfamethoxazole susceptibility remained low compared to other agents.

*H. influenzae* from Ukraine were virtually all β-lactamase negative, and apart from trimethoprim/sulfamethoxazole [61.8% susceptible by CLSI, PK/PD and EUCAST low-dose (0.16 g/0.8 g × 2 oral or IV) and 68.4% susceptible by EUCAST high-dose (0.24 g/0.8 g × 2 oral or IV)], susceptibility to antibiotics was 94.7%–100%, excluding EUCAST low-dose amoxicillin (0.5 g × 3 oral) and amoxicillin/clavulanic acid (0.75–1 g × 3 oral) and cefaclor by PK/PD breakpoints. However, there were differences in susceptibility between CLSI and EUCAST for cefuroxime (0% EUCAST susceptible versus 100% CLSI-susceptible in 2018–21) and macrolides (no EUCAST breakpoints given). No statistical difference in susceptibility by CLSI was observed between 2016–17 and 2018–21.

Except for SOAR data, there are few reports of CA-RTI pathogen susceptibility from Ukraine, but recent data from a clinical study evaluating delafloxacin in Ukraine confirm the high antibiotic susceptibility of *S. pneumoniae*, *H. influenzae* and other bacteria causing community-acquired pneumonia in this country.^16^ It is interesting to observe that low resistance is not always the case for other pathogens in Ukraine. For example, very high levels of multi-drug-resistant tuberculosis (58.1% of previously treated cases) have been recorded in a national survey in Ukraine.^17^ Furthermore, a multicentre study from 2013 to 2015 showed that bloodstream infections in Ukraine had high levels of extended-spectrum beta-lactamases in Enterobacteriaceae (24.8%), methicillin resistance in *Staphylococcus aureus* (38.2%) and carbapenem resistance in *Pseudomonas aeruginosa* and *Acinetobacter baumannii* (33.1% and 63.2%, respectively).^18^

To conclude, high susceptibility was observed to most antibiotics tested, for both *S. pneumoniae* and *H. influenzae* isolates, remaining effectively unchanged since 2011. Continued surveillance of antibiotic susceptibility in Ukraine is imperative to monitor the CA-RTI antimicrobial susceptibility pattern over time.

## Supplementary Material

dkaf287_Supplementary_Data

## Supplementary data

Tables S1 and S2 are available as Supplementary data at *JAC* Online.
