# Supplementary material for: Results from the Survey of Antibiotic Resistance (SOAR) 2018–21 in Ukraine: data based on CLSI, EUCAST (dose-specific) and pharmacokinetic/pharmacodynamic (PK/PD) breakpoints
Source: J Antimicrob Chemother. 2025 Nov 24;80(Suppl 3):iii68–82. doi: 10.1093/jac/dkaf287 (PMC12641123; doi:10.1093/jac/dkaf287)
Supplement: dkaf287_Supplementary_Data [file dkaf287_supplementary_data.docx]

**Results from the Survey of Antibiotic Resistance (SOAR) 2018 – 21 in Ukraine: data based on CLSI, EUCAST (dose-specific) and pharmacokinetic/pharmacodynamic (PK/PD) breakpoints**

**Authors:** Didem TORUMKUNEY^1^, Elena BRATUS^2^, Olena YUVKO^2^, Tetyana PERTSEVA^3^, Ian MORRISSEY^4^, Cristiana Ossaille BELTRAME^5^, Anand MANOHARAN^6*^

**Affiliations:** ^1^GSK, London, UK; ^2^ Dnipropetrovsk State Medical Academy, Diagnostic Center, Dnipropetrovsk, Kyiv, Ukraine; ^3^Dnipropetrovsk State Medical Academy, Dnipro, Ukraine; ^4^Antimicrobial Focus Ltd., Sawbridgeworth, UK; ^5^a Departamento de Microbiologia Médica, Instituto de Microbiologia Paulo de Góes, Universidade Federal do Rio de Janeiro, Rio de Janeiro, Brazil; ^6^Infectious Diseases Medical & Scientific Affairs, GSK, Mumbai, India.

*Corresponding author. E-mail: [anand.x.manoharan@gsk.com](mailto:anand.x.manoharan@gsk.com)

**Running title:** Survey of Antibiotic Resistance (SOAR) in Ukraine in 2018 – 21

**Supplementary Table 1.** MIC distribution data for *S. pneumoniae* isolates (*n* = 64) from Ukraine

|  |  | Number of isolates at MIC (mg/L) | | | | | | | | | | | | | | | | | |
| --- | --- | --- | --- | --- | --- | --- | --- | --- | --- | --- | --- | --- | --- | --- | --- | --- | --- | --- | --- |
| Antimicrobial |  | ≤0.008 | ≤0.015 | 0.015 | 0.03 | 0.06 | 0.12 | ≤0.25 | 0.25 | 0.5 | 1 | 2 | 4 | >4 | 8 | >8 | 16 | >16 |  |
| AMX | N | – | – | 33 | 19 | 1 | 3 | – | 1 | 1 | 2 | 2 | 1 | – | 1 | – | – | – |  |
|  | Cum. % | – | – | 51.6 | 81.3 | 82.8 | 87.5 | – | 89.1 | 90.6 | 93.8 | 96.9 | 98.4 | – | 100 | – | – | – |  |
|  | % | – | – | 51.6 | 29.7 | 1.6 | 4.7 | – | 1.6 | 1.6 | 3.1 | 3.1 | 1.6 | – | 1.6 | – | – | – |  |
| AMC (2:1) | N | 1 | – | 25 | 25 | 2 | 3 | – | – | 3 | 1 | 2 | 1 | – | 1 | – | – | – |  |
|  | Cum. % | 1.6 | – | 40.6 | 79.7 | 82.8 | 87.5 | – | – | 92.2 | 93.8 | 96.9 | 98.4 | – | 100 | – | – | – |  |
|  | % | 1.6 | – | 39.1 | 39.1 | 3.1 | 4.7 | – | – | 4.7 | 1.6 | 3.1 | 1.6 | – | 1.6 | – | – | – |  |
| AMC | N | – | – | – | 23 | 28 | 2 | – | 3 | – | 1 | 2 | 1 | – | 2 | 2 | – | – |  |
| [2 mg/L] | Cum. % | – | – | – | 35.9 | 79.7 | 82.8 | – | 87.5 | – | 89.1 | 92.2 | 93.8 | – | 96.9 | 100 | – | – |  |
|  | % | – | – | – | 35.9 | 43.8 | 3.1 | – | 4.7 | – | 1.6 | 3.1 | 1.6 | – | 3.1 | 3.1 | – | – |  |
| AZM | N | – | 1 | – | 16 | 28 | 3 | – | 1 | – | – | – | 2 | – | – | – | 1 | 12 |  |
|  | Cum. % | – | 1.6 | – | 26.6 | 70.3 | 75.0 | – | 76.6 | – | – | – | 79.7 | – | – | – | 81.3 | 100 |  |
|  | % | – | 1.6 | – | 25.0 | 43.8 | 4.7 | – | 1.6 | – | – | – | 3.1 | – | – | – | 1.6 | 18.8 |  |
| CEC | N | – | – | – | – | 1 | – | – | 3 | 49 | 2 | 2 | – | 7 | – | – | – | – |  |
|  | Cum. % | – | – | – | – | 1.6 | – | – | 6.3 | 82.8 | 85.9 | 89.1 | – | 100 | – | – | – | – |  |
|  | % | – | – | – | – | 1.6 | – | – | 4.7 | 76.6 | 3.1 | 3.1 | – | 10.9 | – | – | – | – |  |
| CDR | N | – | – | – | 2 | 43 | 10 | – | 1 | 2 | 1 | 1 | – | – | 3 | 1 | – | – |  |
|  | Cum. % | – | – | – | 3.1 | 70.3 | 85.9 | – | 87.5 | 90.6 | 92.2 | 93.8 | – | – | 98.4 | 100 | – | – |  |
|  | % | – | – | – | 3.1 | 67.2 | 15.6 | – | 1.6 | 3.1 | 1.6 | 1.6 | – | – | 4.7 | 1.6 | – | – |  |
| CFM | N | – | – | – | – | – | – | 45 | – | 6 | 1 | 5 | 2 | – | 1 | – | 2 | 2 |  |
|  | Cum. % | – | – | – | – | – | – | 70.3 | – | 79.7 | 81.3 | 89.1 | 92.2 | – | 93.8 | – | 96.9 | 100 |  |
|  | % | – | – | – | – | – | – | 70.3 | – | 9.4 | 1.6 | 7.8 | 3.1 | – | 1.6 | – | 3.1 | 3.1 |  |
| CTX | N | 1 | – | 29 | 19 | 4 | 3 | – | 4 | – | 2 | 2 | – | – | – | – | – | – |  |
|  | Cum. % | 1.6 | – | 46.9 | 76.6 | 82.8 | 87.5 | – | 93.8 | – | 96.9 | 100 | – | – | – | – | – | – |  |
|  | % | 1.6 | – | 45.3 | 29.7 | 6.3 | 4.7 | – | 6.3 | – | 3.1 | 3.1 | – | – | – | – | – | – |  |
| CPD | N | – | 2 | – | 43 | 5 | 7 | – | – | 1 | 2 | – | 4 | – | – | – | – | – |  |
|  | Cum. % | – | 3.1 | – | 70.3 | 78.1 | 89.1 | – | – | 90.6 | 93.8 | – | 100 | – | – | – | – | – |  |
|  | % | – | 3.1 | – | 67.2 | 7.8 | 10.9 | – | – | 1.6 | 3.1 | – | 6.3 | – | – | – | – | – |  |
| CTB | N | – | – | – | – | – | – | – | – | – | – | 3 | 43 | – | 6 | – | 2 | 10 |  |
|  | Cum. % | – | – | – | – | – | – | – | – | – | – | 4.7 | 71.9 | – | 81.3 | – | 84.4 | 100 |  |
|  | % | – | – | – | – | – | – | – | – | – | – | 4.7 | 67.2 | – | 9.4 | – | 3.1 | 15.6 |  |
| CRO | N | – | – | 30 | 17 | 5 | 4 | – | 2 | 2 | 2 | 2 | – | – | – | – | – | – |  |
|  | Cum. % | – | – | 46.9 | 73.4 | 81.3 | 87.5 | – | 90.6 | 93.8 | 96.9 | 100 | – | – | – | – | – | – |  |
|  | % | – | – | 46.9 | 26.6 | 7.8 | 6.3 | – | 3.1 | 3.1 | 3.1 | 3.1 | – | – | – | – | – | – |  |
| CXM | N | – | – | 14 | 30 | 4 | 3 | – | 3 | 2 | 2 | 1 | 3 | – | 1 | 1 | – | – |  |
|  | Cum. % | – | – | 21.9 | 68.8 | 75.0 | 79.7 | – | 84.4 | 87.5 | 90.6 | 92.2 | 96.9 | – | 98.4 | 100 | – | – |  |
|  | % | – | – | 21.9 | 46.9 | 6.3 | 4.7 | – | 4.7 | 3.1 | 3.1 | 1.6 | 4.7 | – | 1.6 | 1.6 | – | – |  |
| CLR | N | – | 27 | – | 22 | – | – | – | – | – | 1 | 1 | 1 | – | – | – | – | 12 |  |
|  | Cum. % | – | 42.2 | – | 76.6 | – | – | – | – | – | 78.1 | 79.7 | 81.3 | – | – | – | – | 100 |  |
|  | % | – | 42.2 | – | 34.4 | – | – | – | – | – | 1.6 | 1.6 | 1.6 | – | – | – | – | 18.8 |  |
| DOX | N | – | – | – | 2 | 36 | 9 | – | – | 2 | 1 | 1 | 3 | 10 | – | – | – | – |  |
|  | Cum. % | – | – | – | 3.1 | 59.4 | 73.4 | – | – | 76.6 | 78.1 | 79.7 | 84.4 | 100 | – | – | – | – |  |
|  | % | – | – | – | 3.1 | 56.3 | 14.1 | – | – | 3.1 | 1.6 | 1.6 | 4.7 | 15.6 | – | – | – | – |  |
| ERY | N | – | 16 | – | 30 | 3 | – | – | – | – | – | 2 | 1 | – | – | – | – | 12 |  |
|  | Cum. % | – | 25.0 | – | 71.9 | 76.6 | – | – | – | – | – | 79.7 | 81.3 | – | – | – | – | 100 |  |
|  | % | – | 25.0 | – | 46.9 | 4.7 | – | – | – | – | – | 3.1 | 1.6 | – | – | – | – | 18.8 |  |
| LVX | N | – | – | – | – | – | – | – | – | 10 | 46 | 8 | – | – | – | – | – | – |  |
|  | Cum. % | – | – | – | – | – | – | – | – | 15.6 | 87.5 | 100 | – | – | – | – | – | – |  |
|  | % | – | – | – | – | – | – | – | – | 15.6 | 71.9 | 12.5 | – | – | – | – | – | – |  |
| MXF | N | – | – | – | – | 27 | 36 | – | 1 | – | – | – | – | – | – | – | – | – |  |
|  | Cum. % | – | – | – | – | 42.2 | 98.4 | – | 100 | – | – | – | – | – | – | – | – | – |  |
|  | % | – | – | – | – | 42.2 | 56.3 | – | 1.6 | – | – | – | – | – | – | – | – | – |  |
| PEN | N | 1 | – | 38 | 9 | 5 | 3 | – | 2 | 2 | – | 3 | 1 | – | – | – | – | – |  |
|  | Cum. % | 1.6 | – | 60.9 | 75.0 | 82.8 | 87.5 | – | 90.6 | 93.8 | – | 98.4 | 100 | – | – | – | – | – |  |
|  | % | 1.6 | – | 59.4 | 14.1 | 7.8 | 4.7 | – | 3.1 | 3.1 | – | 4.7 | 1.6 | – | – | – | – | – |  |
| TET | N | – | – | – | – | 2 | 24 | – | 17 | 4 | – | – | 1 | 16 | – | – | – | – |  |
|  | Cum. % | – | – | – | – | 3.1 | 40.6 | – | 67.2 | 73.4 | – | – | 75.0 | 100 | – | – | – | – |  |
|  | % | – | – | – | – | 3.1 | 37.5 | – | 26.6 | 6.3 | – | – | 1.6 | 25.0 | – | – | – | – |  |
| SXT | N | – | – | – | – | – | 14 | – | 17 | 7 | 13 | – | 4 | – | 6 | 3 | – | – |  |
|  | Cum. % | – | – | – | – | – | 21.9 | – | 48.4 | 59.4 | 79.7 | – | 85.9 | – | 95.3 | 100 | – | – |  |
|  | % | – | – | – | – | – | 21.9 | – | 26.6 | 10.9 | 20.3 | – | 6.3 | – | 9.4 | 4.7 | – | – |  |

–, not applicable; AMC, amoxicillin/clavulanic acid; AMP, ampicillin; AMX, amoxicillin; AZM, azithromycin; CDR, cefdinir; CEC, cefaclor; CFM, cefixime; CLR, clarithromycin; CPD, cefpodoxime; CRO, ceftriaxone; CTB, ceftibuten; CTX, cefotaxime; Cum., cumulative; CXM, cefuroxime; LVX, levofloxacin; MXF, moxifloxacin; SXT, trimethoprim/sulfamethoxazole; TET, tetracycline.

Bold vertical bars in table correspond to the CLSI-susceptible breakpoints

**Supplementary Table 2.** MIC distribution data for *H. influenzae* isolates (*n* = 76) from Ukraine

|  |  |  | | |  |  | | Number of isolates at MIC (mg/L) | | | | | | | | | | | | | | | | | | | | | |
| --- | --- | --- | --- | --- | --- | --- | --- | --- | --- | --- | --- | --- | --- | --- | --- | --- | --- | --- | --- | --- | --- | --- | --- | --- | --- | --- | --- | --- | --- |
| Antimicrobial |  | | ≤0.001 | ≤0.002 | | 0.002 | ≤0.004 | | 0.004 | ≤0.008 | 0.008 | ≤0.015 | 0.015 | 0.03 | 0.06 | 0.12 | ≤0.25 | 0.25 | 0.5 | 1 | 2 | 4 | 8 | >8 | 16 | 32 | 64 | 128 |  |
| AMX | N | | – | – | | – | – | | – | – | – | – | – | – | – | 2 | – | 41 | 20 | 6 | 3 | – | – | – | – | 2 | 2 | – |  |
|  | Cum. % | | – | – | | – | – | | – | – | – | – | – | – | – | 2.6 | – | 56.6 | 82.9 | 90.8 | 94.7 | – | – | – | – | 97.4 | 100 | – |  |
|  | % | | – | – | | – | – | | – | – | – | – | – | – | – | 2.6 | – | 53.9 | 26.3 | 7.9 | 3.9 | – | – | – | – | 2.6 | 2.6 | – |  |
| AMC (2:1) | N | | – | – | | – | – | | – | – | – | – | – | – | – | 2 | – | 39 | 22 | 10 | 3 | – | – | – | – | – | – | – |  |
|  | Cum. % | | – | – | | – | – | | – | – | – | – | – | – | – | 2.6 | – | 53.9 | 82.9 | 96.1 | 100 | – | – | – | – | – | – | – |  |
|  | % | | – | – | | – | – | | – | – | – | – | – | – | – | 2.6 | – | 51.3 | 28.9 | 13.2 | 3.9 | – | – | – | – | – | – | – |  |
| AMC | N | | – | – | | – | – | | – | – | – | – | – | – | – | 11 | – | 38 | 21 | 3 | 3 | – | – | – | – | – | – | – |  |
| [2 mg/L] | Cum. % | | – | – | | – | – | | – | – | – | – | – | – | – | 14.5 | – | 64.5 | 92.1 | 96.1 | 100 | – | – | – | – | – | – | – |  |
|  | % | | – | – | | – | – | | – | – | – | – | – | – | – | 14.5 | – | 50.0 | 27.6 | 3.9 | 3.9 | – | – | – | – | – | – | – |  |
| AMP | N | | – | – | | – | – | | – | – | – | – | – | – | – | 43 | – | 20 | 5 | 4 | – | – | – | – | – | – | 3 | 1 |  |
|  | Cum. % | | – | – | | – | – | | – | – | – | – | – | – | – | 56.6 | – | 82.9 | 89.5 | 94.7 | – | – | – | – | – | – | 98.7 | 100 |  |
|  | % | | – | – | | – | – | | – | – | – | – | – | – | – | 56.6 | – | 26.3 | 6.6 | 5.3 | – | – | – | – | – | – | 3.9 | 1.3 |  |
| AZM | N | | – | – | | – | – | | – | – | – | – | – | – | – | – | – | 4 | 38 | 31 | 3 | – | – | – | – | – | – | – |  |
|  | Cum. % | | – | – | | – | – | | – | – | – | – | – | – | – | – | – | 5.3 | 55.3 | 96.1 | 100 | – | – | – | – | – | – | – |  |
|  | % | | – | – | | – | – | | – | – | – | – | – | – | – | – | – | 5.3 | 50.0 | 40.8 | 3.9 | – | – | – | – | – | – | – |  |
| CEC | N | | – | – | | – | – | | – | – | – | – | – | – | – | – | 1 | – | 8 | 36 | 20 | 6 | 4 | – | 1 | – | – | – |  |
|  | Cum. % | | – | – | | – | – | | – | – | – | – | – | – | – | – | 1.3 | – | 11.8 | 59.2 | 85.5 | 93.4 | 98.7 | – | 100 | – | – | – |  |
|  | % | | – | – | | – | – | | – | – | – | – | – | – | – | – | 1.3 | – | 10.5 | 47.4 | 26.3 | 7.9 | 5.3 | – | 1.3 | – | – | – |  |
| CDR | N | | – | – | | – | – | | – | – | – | – | – | – | – | 28 | – | 38 | 5 | 5 | – | – | – | – | – | – | – | – |  |
|  | Cum. % | | – | – | | – | – | | – | – | – | – | – | – | – | 36.8 | – | 86.8 | 93.4 | 100 | – | – | – | – | – | – | – | – |  |
|  | % | | – | – | | – | – | | – | – | – | – | – | – | – | 36.8 | – | 50.0 | 6.6 | 6.6 | – | – | – | – | – | – | – | – |  |
| CFM | N | | – | – | | – | – | | – | 1 | – | – | 33 | 37 | 3 | – | – | 2 | – | – | – | – | – | – | – | – | – | – |  |
|  | Cum. % | | – | – | | – | – | | – | 1.3 | – | – | 44.7 | 93.4 | 97.4 | – | – | 100 | – | – | – | – | – | – | – | – | – | – |  |
|  | % | | – | – | | – | – | | – | 1.3 | – | – | 43.4 | 48.7 | 3.9 | – | – | 2.6 | – | – | – | – | – | – | – | – | – | – |  |
| CTX | N | | – | 2 | | – | – | | 1 | – | 42 | – | 22 | 8 | 1 | – | – | – | – | – | – | – | – | – | – | – | – | – |  |
|  | Cum. % | | – | 2.6 | | – | – | | 3.9 | – | 59.2 | – | 88.2 | 98.7 | 100 | – | – | – | – | – | – | – | – | – | – | – | – | – |  |
|  | % | | – | 2.6 | | – | – | | 1.3 | – | 55.3 | – | 28.9 | 10.5 | 1.3 | – | – | – | – | – | – | – | – | – | – | – | – | – |  |
| CPD | N | | – | – | | – | – | | – | – | – | 3 | – | 36 | 28 | 4 | – | 5 | – | – | – | – | – | – | – | – | – | – |  |
|  | Cum. % | | – | – | | – | – | | – | – | – | 3.9 | – | 51.3 | 88.2 | 93.4 | – | 100 | – | – | – | – | – | – | – | – | – | – |  |
|  | % | | – | – | | – | – | | – | – | – | 3.9 | – | 47.4 | 36.8 | 5.3 | – | 6.6 | – | – | – | – | – | – | – | – | – | – |  |
| CTB | N | | – | – | | – | – | | – | – | – | – | – | 28 | 37 | 4 | – | 5 | – | 1 | 1 | – | – | – | – | – | – | – |  |
|  | Cum. % | | – | – | | – | – | | – | – | – | – | – | 36.8 | 85.5 | 90.8 | – | 97.4 | – | 98.7 | 100 | – | – | – | – | – | – | – |  |
|  | % | | – | – | | – | – | | – | – | – | – | – | 36.8 | 48.7 | 5.3 | – | 6.6 | – | 1.3 | 1.3 | – | – | – | – | – | – | – |  |
| CRO | N | | 2 | – | | 31 | – | | 32 | – | 7 | – | 4 | – | – | – | – | – | – | – | – | – | – | – | – | – | – | – |  |
|  | Cum. % | | 2.6 | – | | 43.4 | – | | 85.5 | – | 94.7 | – | 100 | – | – | – | – | – | – | – | – | – | – | – | – | – | – | – |  |
|  | % | | 2.6 | – | | 40.8 | – | | 42.1 | – | 9.2 | – | 5.3 | – | – | – | – | – | – | – | – | – | – | – | – | – | – | – |  |
| CXM | N | | – | – | | – | – | | – | – | – | – | – | – | – | – | – | 19 | 38 | 13 | 5 | 1 | – | – | – | – | – | – |  |
|  | Cum. % | | – | – | | – | – | | – | – | – | – | – | – | – | – | – | 25.0 | 75.0 | 92.1 | 98.7 | 100 | – | – | – | – | – | – |  |
|  | % | | – | – | | – | – | | – | – | – | – | – | – | – | – | – | 25.0 | 50.0 | 17.1 | 6.6 | 1.3 | – | – | – | – | – | – |  |
| CLR | N | | – | – | | – | – | | – | – | – | – | – | – | – | – | – | – | – | – | 7 | 43 | 26 | – | – | – | – | – |  |
|  | Cum. % | | – | – | | – | – | | – | – | – | – | – | – | – | – | – | – | – | – | 9.2 | 65.8 | 100 | – | – | – | – | – |  |
|  | % | | – | – | | – | – | | – | – | – | – | – | – | – | – | – | – | – | – | 9.2 | 56.6 | 34.2 | – | – | – | – | – |  |
| LVX | N | | – | – | | – | – | | – | – | 4 | – | 65 | 4 | – | 1 | – | – | – | – | – | – | 2 | – | – | – | – | – |  |
|  | Cum. % | | – | – | | – | – | | – | – | 5.3 | – | 90.8 | 96.1 | – | 97.4 | – | – | – | – | – | – | 100 | – | – | – | – | – |  |
|  | % | | – | – | | – | – | | – | – | 5.3 | – | 85.5 | 5.3 | – | 1.3 | – | – | – | – | – | – | 2.6 | – | – | – | – | – |  |
| MXF | N | | – | – | | – | 1 | | – | – | 8 | – | 47 | 16 | 1 | 1 | – | – | – | – | – | – | 2 | – | – | – | – | – |  |
|  | Cum. % | | – | – | | – | 1.3 | | – | – | 11.8 | – | 73.7 | 94.7 | 96.1 | 97.4 | – | – | – | – | – | – | 100 | – | – | – | – | – |  |
|  | % | | – | – | | – | 1.3 | | – | – | 10.5 | – | 61.8 | 21.1 | 1.3 | 1.3 | – | – | – | – | – | – | 2.6 | – | – | – | – | – |  |
| TET | N | | – | – | | – | – | | – | – | – | – | – | – | – | – | – | 35 | 41 | – | – | – | – | – | – | – | – | – |  |
|  | Cum. % | | – | – | | – | – | | – | – | – | – | – | – | – | – | – | 46.1 | 100 | – | – | – | – | – | – | – | – | – |  |
|  | % | | – | – | | – | – | | – | – | – | – | – | – | – | – | – | 46.1 | 53.9 | – | – | – | – | – | – | – | – | – |  |
| SXT | N | | – | – | | – | – | | – | – | – | – | – | 2 | 26 | 14 | – | 5 | – | 5 | 4 | 4 | 13 | 3 | – | – | – | – |  |
|  | Cum. % | | – | – | | – | – | | – | – | – | – | – | 2.6 | 36.8 | 55.3 | – | 61.8 | – | 68.4 | 73.7 | 78.9 | 96.1 | 100 | – | – | – | – |  |
|  | % | | – | – | | – | – | | – | – | – | – | – | 2.6 | 34.2 | 18.4 | – | 6.6 | – | 6.6 | 5.3 | 5.3 | 17.1 | 3.9 | – | – | – | – |  |

–, not applicable; AMC, amoxicillin/clavulanic acid; AMP, ampicillin; AMX, amoxicillin; AZM, azithromycin; CDR, cefdinir; CEC, cefaclor; CFM, cefixime; CLR, clarithromycin; CPD, cefpodoxime; CRO, ceftriaxone; CTB, ceftibuten; CTX, cefotaxime; Cum., cumulative; CXM, cefuroxime; LVX, levofloxacin; MXF, moxifloxacin; SXT, trimethoprim/sulfamethoxazole; TET, tetracycline.

Bold vertical bars in table correspond to the CLSI-susceptible breakpoints.
